# Perceptions of Malaria in Pregnancy and Acceptability of Preventive Interventions among Mozambican Pregnant Women: Implications for Effectiveness of Malaria Control in Pregnancy

**DOI:** 10.1371/journal.pone.0086038

**Published:** 2014-02-03

**Authors:** Helena Boene, Raquel González, Anifa Valá, Maria Rupérez, César Velasco, Sónia Machevo, Charfudin Sacoor, Esperança Sevene, Eusébio Macete, Clara Menéndez, Khátia Munguambe

**Affiliations:** 1 Centro de Investigação em Saúde da Manhiça, Manhiça, Mozambique; 2 Barcelona Centre for International Health Research, Hospital Clinic - Universitat de Barcelona, Barcelona, Spain; 3 Direcção Nacional de Saúde Pública, Ministério da Saúde, Maputo, Mozambique; 4 Universidade Eduardo Mondlane, Faculdade de Medicina, Maputo, Mozambique; Tulane University School of Public Health and Tropical Medicine, United States of America

## Abstract

**Background:**

Intermittent Preventive Treatment (IPTp) and insecticide treated nets (ITNs) are recommended malaria in pregnancy preventive interventions in sub-Saharan Africa. Despite their cost-effectiveness and seemingly straight-forward delivery mechanism, their uptake remains low. We aimed at describing perceptions of pregnant women regarding malaria and the recommended prevention interventions to understand barriers to uptake and help to improve their effectiveness.

**Methods and findings:**

We used mixed methods to collect data among 85 pregnant women from a rural area of Southern Mozambique. Information was obtained through observations, in-depth interviews, and focused ethnographic exercises (Free-listing and Pairwise comparisons). Thematic analysis was performed on qualitative data. Data from focused ethnographic exercises were summarized into frequency distribution tables and matrices. Malaria was not viewed as a threat to pregnancy. Participants were not fully aware of malaria- associated adverse maternal and birth outcomes. ITNs were the most preferred and used malaria preventive intervention, while IPTp fell between second and third. Indoor Residual Spraying (IRS) was the least preferred intervention.

**Conclusions:**

Low awareness of the risks and adverse consequences of malaria in pregnancy did not seem to affect acceptability or uptake to the different malaria preventive interventions in the same manner. Perceived convenience, the delivery approach, and type of provider were the key factors. Pregnant women, through antenatal care (ANC) services, can be the vehicles of ITN distribution in the communities to maximise overall ITN coverage. There is a need to improve knowledge about neonatal health and malaria to improve uptake of interventions delivered through channels other than the health facility.

## Introduction

Pregnant women are the main adult group at risk of malaria in sub-Saharan Africa, where approximately 25 million pregnancies are exposed to the infection and an estimated 10,000 maternal deaths attributable to malaria occur every year [1]. To control malaria in pregnancy the World Health Organisation (WHO) recommends IPTP with sulfadoxine-pyrimetamine (SP), use of insecticide treated nets (ITNs), and effective treatment of malaria episodes [2]. Despite the fact that both IPTp with SP and ITNs are highly cost-effective in improving maternal and infant health [3], and have been rolled out for many years in several malaria endemic countries, their coverage is still unacceptably low in sub-Saharan Africa [4].

Several factors may explain the low uptake, and hence low effectiveness of preventive interventions for malaria in pregnancy, such as, limited access to ANC services, health system factors which include drugs and ITN stock outs, health professionals' attitudes and practices, low patient adherence, or community attitudes towards one intervention among others [5], [6].

Anthropological studies have suggested variations in individual and community's malaria-related beliefs according to their socio-cultural, educational, economic, and environmental contexts and backgrounds, which in turn affect malaria prevention outcomes [7], [8]. For example, in Malawi it has been reported that local taboos prohibiting the ingestion of bitter substances during pregnancy posed limitations on the acceptability of potential anti-malarial drugs for IPTp [7]. In addition, the belief that drugs lead to miscarriage or to difficult labour due to large-sized babies have also been suggested to be related to the low uptake of preventive drugs for malaria in pregnancy [5].

It is recognized that there has been more focus on quantitative data collection approaches on perceptions of malaria in pregnancy and acceptability of preventive interventions compared to qualitative or mixed methods [8]. Moreover, studies on acceptability of malaria preventive interventions often examine them in isolation from other interventions. Therefore there is a need to better understand women's perceptions, acceptability and adherence to these interventions when they are integrated with other health services offered to them, such as ANC services [5], [9].

In Mozambique, since 2006 IPTp is administered under directly observed therapy, and ITNs are delivered to pregnant women free of charge through ANC [10]. However, the average country uptake of at least two IPTp doses is still only 23% and the proportion of households with pregnant women who have at least one ITN is about 19% [11]. There is a lack of information about the acceptability of specific malaria preventive interventions among Mozambican pregnant women. A context-specific analysis might provide insights as to what extent factors such as perceptions and beliefs, may be related to the low uptake of malaria preventive interventions.

This study aimed at describing the perceptions and behaviours of pregnant women in relation to malaria and the acceptability of currently recommended malaria preventive interventions. This information may serve to understand the barriers that affect the uptake of malaria preventive interventions in pregnancy and help to improve their effectiveness.

## Materials and Methods

### Study site and population

The study was conducted in Manhiça, southern Mozambique, located 80 Km north of Maputo city. The district covers an area of 2,360 Km2 and its population in 2007 was around 160,000 inhabitants, of which 23% comprised women of reproductive age [12]. Malaria transmission is perennial, with moderate seasonality that peaks during the rainy season (from November to March), and mainly due to Plasmodium falciparum [13]. The prevalence of HIV in women attending the ANC clinic was 29.4% in 2010 [14].

The population of Manhiça belongs mainly to the Changana ethnic group. They are mostly subsistence farmers, informal traders, employees of two local sugar estates, and migrant labours in South Africa [15]. Adult illiteracy rate is 71%, being more prevalent among women [15].

Around 92.000 inhabitants are under continuous follow-up through a Demographic Surveillance System (DSS), which covers an area of around 500 Km^2^ (also known as the Demographic Surveillance Area) and routinely registers pregnancies, births, deaths, and migrations. During 2009, the DSS registered 2,539 new pregnancies (unpublished data, Manhiça DSS 2009), and around 2,000 women attended ANC visits (unpublished data, MDH 2009).

The ANC clinic at the Manhiça District Hospital (MDH) offers maternal and child health treatment and preventive services that include screening and treatment of syphilis, anaemia, and urinary tract infections, IPTp with SP (administered 3 times during pregnancy), ITNs distribution, antihelmintic, ferrous sulphate and folate tablets, tetanus toxoid vaccination, and prevention of mother to child transmission (PMTCT) of HIV with administration of antiretrovirals [16].

For malaria prevention, in addition, indoor residual spraying (IRS) is carried out in yearly rounds in the community.

### Study design and data collection

This was a mixed method study, generating qualitative and quantitative data. Specifically, standard qualitative data collection approaches namely observation of ANC clinic activities and in-depth interviews (IDIs) with pregnant women were combined with focused ethnographic study (FES) data collection approaches [17], [18]. Focused ethnographic studies address some of the limitations of the classic ethnographic design, by making use of a package of exercises that can be applied during the course of individual or group interviews [18], [19]. The exercises used in this study were Free-listing and Pairwise comparison. The exercises help to swiftly generate, organize and categorize insights regarding local perceptions and behaviours and how these are shaped by cultural norms.

Data was collected by a trained female Mozambican social scientist with experience in conducting qualitative studies (HB) assisted by a local female interviewer (RP), who was specifically trained for this study. Both of them are fluent in Portuguese and Changana languages. The majority of interviews and FES exercises were conducted in Changana and very few were conducted in Portuguese. Choice of language was determined by participants' convenience.

Through in-depth interviews (Form S1), perceptions about malaria in pregnancy, and the use and acceptability of conventional and traditional malaria preventive interventions were explored. The interviews targeted both women attending ANC and women who had not attended any ANC visit during their current pregnancy. Participants not attending ANC were involved in a single interview at home, while ANC attendants were expected to be involved in three interviews. The first interview with ANC attendants was conducted at the health facility immediately after the administration of the first IPTp dose. For the second and third interviews women were followed up at home, up to two weeks after administration of the second IPTp dose, and up to 24 weeks after the end of the pregnancy respectively. While the first and second interviews focused on aspects related to pregnant women's perceptions and experiences with the different interventions offered to them during ANC, the third interview dealt, in addition, with their overall degree of satisfaction with regards to such interventions in the light of the pregnancy outcome, and any other issues that the interviewer had not managed to address during the previous interviews. Interviewing the participants several times and outside the health facility environment allowed rapport building between the interviewer and the participant.

During the IDIs with ANC attendants, Free-listing and Pairwise comparison exercises were conducted as part of the Focused Ethnographic Study component. Through the Free-listing exercise (Form S2), a list of perceived and experienced health problems and complaints during pregnancy was generated, through an open-ended question, at the start of each IDI. The interviewer then probed about perceived causes and consequences of each of the identified health problems both for the mother and the foetus. The exercise ended by the interviewer revisiting each of the mentioned complaints and discussing the perceived severity and the perceived risk of contracting the illness.

Pairwise comparison consisted in the use of pictorial vignettes representing malaria preventive interventions and other interventions delivered through ANC. First, the vignettes were presented to the participants all at once, and an agreement was reached between the participant and the interviewer as to which intervention each vignette represented. The participant was asked to organize the vignettes from the most preferred to the least preferred intervention. The interviewer used a data extraction form to register the preferences (Form S3). Secondly, the vignettes were presented to the participant in pairs, i.e., each vignette was matched one-on-one with another vignette at a time, encouraging the participant to think about the relative advantages and disadvantages of each of the presented interventions. For each pair, the participant was asked to elect the favourite one, and a discussion followed regarding the reasons for the choice.

Lastly, observations were carried out at the ANC clinics, both in the waiting and consultation rooms. The latter generated information about the participants' interaction with health care providers, as well as on their reactions to the procedures offered to them. It also provided insights about the possible contextual factors impeding or facilitating the implementation and acceptability of the interventions.

### Recruitment

This study took place from September 2010 to November 2011. Two groups of participants were recruited. One group comprised pregnant women attending the first ANC visit and willing to participate in the qualitative study. Every day, the ANC nurse recruited the first two patients who fulfilled the inclusion criteria. The social scientist then asked for consent and arranged for the appropriate time and place for an initial interview.

To capture the views and opinions of those who had not had access to ANC services, a second group of participants was recruited, consisting of randomly selected pregnant women identified through the DSS. Every day, two women were recruited by the DSS fieldworker who annotated the directions of the household and possible dates for an initial interview.

A minimum sample size of 20 per group was foreseen in order to fulfil the study objectives. A final sample size of 85 women was obtained based on the saturation point, which is reached when the inclusion of new participants is suspended due to redundancy in the data being generated [20].

The number of observations was also determined by saturation, giving a total of 30 observations conducted at the ANC clinic over a period of six months.

### Ethics statement

Ethical approval for this study was granted by the National Health Bioethics Committee in Mozambique (IRB nr. 00002657).

Written informed consent was sought from all participants. Among the participants of this study we included a subgroup of 11 pregnant women between the ages of 15 and 18, reflecting the range of ages of pregnant women at risk of malaria in the study area. Regarding participation of these women in the study, a waiver for parental consent was given by the IRB because they are considered to be *mature minors* who make autonomous decisions regarding their pregnancy and foetus. In fact, the majority of them were either married or not living with their parents. Moreover, culturally, the likelihood of an undisclosed pregnancy in the first trimester is high, therefore parental permission, which implied disclosure of pregnancy status, would pose more social harm to the participants than the participation in the study without parental permission. Participation of this group of women was crucial because they are the group most at risk of malaria-related pregnancy adverse consequences. Exclusion of this group from studies like this may deny benefits to this group on the long run.

### Data management and analysis

All data analysis was performed using MS Excel [21] and Paired Comparison Worksheet [22].

For the data generated through the Free-listing exercise, all the illnesses that were identified by the participants were sorted in decreasing order of frequency of being mentioned. Further, for each illness, the perceived symptoms, preventive measures, and known treatment options were tabulated according to the frequency of being mentioned [17], [18], [23].

Analysis of Pairwise comparisons was performed in two steps. First, for the component of the Pairwise comparison whereby each participant was asked to rank their preference among the seven interventions presented to them all at once, the interventions were tabulated according to the frequency in which these interventions were elected as 1^st^, 2^nd^, …n^th^ choice (n = 7). Secondly, for the component of the Pairwise comparison whereby participants were asked to choose their preference between interventions that were presented in pairs, each intervention obtained one to three points according to the difference in the frequency mentioned compared to the alternative, and zero points for a tie. Interventions were then ranked according to the total number of points obtained after the scores of all possible comparison pairs were added [24].

The in-depth interviews and the discussions held during the FES exercises were digitally recorded and transcribed *verbatim* and field notes were taken during observations. Transcriptions and field notes were read and data were extracted and tabulated according to three key themes: (i) perceptions and knowledge of malaria in pregnancy; (ii) knowledge and acceptability of ANC services; (iii) acceptability and use of malaria preventive interventions in general and IPTp in particular.

## Results

### Characteristics of respondents

A total of 85 pregnant women participated in the study, of which 21 were recruited at the community and 64 at the ANC. Women recruited at the community participated in only one interview because as they were not ANC service users, there were no anticipated further issues to discuss with them after the first interview. Of the 64 women recruited at the ANC, all participated in the first interview (immediately after the 1^st^ IPTp dose), 36 participated in the second (four to six weeks after the 1^st^ dose, assuming that the 2^nd^ dose would have been given four weeks after the 1^st^ dose and adding a window period of two weeks to allow for tracking of participants at home), and 54 in the third (up to 24 weeks after the end of the pregnancy). The reasons for lower participation in the second and third interviews are presented in Table 1. The majority of losses to follow-up in the second round of interviews were because their first dose was administered very close to the due date, which implied that they had delivered when they were visited at home for the second round of interviews and therefore had to skip to the final interview, ending up with only two interviews.

**Table 1:** Reasons for non-participation in rounds 2 and 3 of interviews (n = 64).

Interview round	Number not interviewed	Reasons for not being interviewed	
2	28	Unavailable	1
		Had given birth	27
3	10	Unavailable	3
		Refused	3
		Internal migration	4

The participants were between 15 and 40 years of age. Most women had primary education level (65%), 21% had secondary education level, and the majority were housewives (69%). Sixty percent of the respondents were in a marital union. Detailed socio-demographic characteristics are presented in Table 2.

**Table 2:** Socio-demographic characteristics of respondents.

Characteristic	Frequency (%)
*Age*	
15–24	52 (61)
25–34	26 (31)
35–44	7 (8)
*Education level*	
No formal education	11 (13)
Primary	56 (66)
Secondary	18 (21)
*Marital Status*	
Married/Union	51 (60)
Casual relationship	9 (11)
Separated/Divorced	25 (29)
*Occupation*	
Housewife	59 (70)
Student	12 (14)
Vendor	5 (6)
Subsistence Farmer	4 (5)
Formally employed	5 (5)
*Parity*	
0 Children	31 (36)
1–2 Children	33 (38)
≥3 Children	22 (26)
*Gestational age at recruitment*	
≤3 months	7 (8)
4 to 6 months	58 (68)
7 to 9 months	20 (24)
Total	85 (100)

### Perceptions of malaria in pregnancy

When responding to the open-ended question about perceived illnesses in pregnancy, malaria (locally referred as *Dzedzedze*) was the most frequently mentioned health problem in pregnancy, followed by abdominal, back and body pains. Headache, fever, chills, and joint pains were mentioned as the main symptoms of malaria in pregnancy (Table 3). The majority of pregnant women (74%), perceived the mosquito bite as the main mode of malaria transmission. Other ways of transmission were mentioned, such as unhygienic backyards and heat.

**Table 3:** Perceived symptoms of illnesses during pregnancy.

Illness	Symptoms*
Malaria	**Headache, fever, chills, joint pains**, abdominal pain, lack of appetite, nausea, palpitations, asthenia, dizziness, flu symptoms, hot flushes
Abdominal pain	**Pain**
Swollen feet	#
Joint pain	#
Vomiting	#
Womb pain	#
Vaginal discharge	#
Back and body pains	Pain
Cough	Pain
Weakness	#

* Perceived principal symptoms in bold type.

# No description of symptoms because according to the respondents the illness name was self explanatory of the symptoms.

More than half (65%) of the participants reported that pregnant women and children were the groups most at risk for the infection.

*“Pregnant women are those who catch malaria more easily. Even among those who are not pregnant, there are those who catch malaria more easily. But it happens more often to us, pregnant women, because we can catch any type of disease easily, because of our state [being pregnant]”*. 24 year old pregnant women, IDI2

In the view of the participants, the increased risk of malaria in pregnant women resulted from sharing their protection with the unborn child, and therefore, being more fragile and susceptible to malaria.

In contrast to their knowledge about pregnant women's vulnerability to malaria, 58% of participants did not consider themselves at risk or ignored the risk of malaria because they felt protected by ITNs and IPTp.

*“No, [I don't feel at risk of getting malaria] because every day I sleep under a net, and I take the tablets in the hospital, the ones they said are to treat malaria”*. - 24 years old pregnant women, IDI1

Fifty-four women believed that malaria in pregnancy had adverse consequences. When asked through an open-ended question about the specific deleterious effects of malaria, the majority only referred to the consequences for the pregnant woman (maternal death, and hallucinations), very few mentioned consequences for the foetus or the baby (miscarriage, stillbirth, congenital malaria, prematurity) having only one respondent indicated low birth weight.

### Preferences of malaria preventive interventions in the context of ANC routine activities

Regarding pregnant women's preferences of the different interventions against all others, Table 4 presents the frequency distribution of interventions among those elected as first, second, and subsequently up to seventh choice. Of note, 62.5% of the first choices were ITNs, followed by IPTp which amounted to 12.5% of the first choices. Nine percent of the first choices were iron supplements and HIV PMTCT. The interventions which were least elected as the first choice were tetanus toxoid vaccine (TT vaccine), antihelmintics, and IRS. The top second choices were IPTp and iron supplements and the bottom three choices were antihelmintics, IRS and HIV PMTCT.

**Table 4:** Distribution of malaria preventive interventions and other interventions delivered through ANC among each choice.

	Percentage distribution of interventions among each choice						
Prevention interventions	1^st^ choice	2^nd^ choice	3^rd^ choice	4^th^ choice	5^th^ choice	6^th^ choice	7^th^ choice
ITN	62.5	10.5	7.6	7.6	3.8	1.3	0.0
IPTp	12.5	25.6	15.2	10.1	10.3	11.4	10.7
IRS	1.1	11.6	17.7	13.9	16.7	19.0	22.7
Iron supplements	9.1	23.3	21.5	25.3	9.0	13.9	4.0
Tetanus vaccine	3.4	15.1	25.3	24.1	23.1	6.3	5.3
HIV PMTCT	9.1	9.3	7.6	10.1	19.2	20.3	21.3
Antihelmintic	2.3	4.7	5.1	8.9	17.9	27.8	36.0
**Total**	100	100	100	100	100	100	100

Table 5 shows, for each intervention, the proportion of times elected as favourite, second choice and subsequently until the last choice. In terms of malaria interventions, ITNs were by far mostly mentioned as first choice (69%). IPTp was mostly mentioned as second choice (28%), but also and less frequently, elected as first and third choice (15% and 14% respectively). IRS was mostly mentioned between the third and the last three choices and hardly elected as the first choice (1.2%). In terms of other interventions, iron supplements were mostly elected between the second, third and fourth choices, and tetanus vaccine was more skewed towards the third, fourth and fifth choices. Both antihelmintics and HIV PMTCT were mostly elected among the last three choices.

**Table 5:** Distribution of the choices made for each malaria preventive interventions and other interventions delivered through ANC.

	Percentage distribution of the choices among each intervention							
Prevention interventions	1^st^ choice	2^nd^ choice	3^rd^ choice	4^th^ choice	5^th^ choice	6^th^ choice	7^th^ choice	Total
ITN	68.8	11.3	7.5	7.5	3.8	1.3	0.0	100
IPTp	14.1	28.2	15.4	10.3	10.3	11.5	10.3	100
IRS	1.2	12.3	17.3	13.6	16.0	18.5	21.0	100
Iron supplements	9.3	23.3	19.8	23.3	8.1	12.8	3.5	100
Tetanus vaccine	3.7	15.9	24.4	23.2	22.0	6.1	4.9	100
HIV PMTCT	10.4	10.4	7.8	10.4	19.5	20.8	20.8	100
Antihelmintic	2.5	5.0	5.0	8.8	17.5	27.5	33.8	100

Table 6 shows the comparison of each intervention against one alternative at a time. This comparison revealed that ITNs (with a total score of 14 points) were relatively more important than all other interventions except for HIV PMTCT. IPTp (with 5 points) was only more important than IRS and antihelmintics, which had been revealed to be the two least favourite interventions in the first step of the Pairwise comparison exercise (Tables 4 and 5). IRS (0 points) was consistently less preferred than all other interventions. Among the other interventions delivered through ANC, HIV PMTCT (8 points) and tetanus toxoid vaccine (7 points) were the relatively most important ones compared to each of the other interventions.

**Table 6:** Pairwise comparison matrix with scores of each intervention matched against all others.

	A: ITNs	B: IPTp	C: IRS	D: Iron	E: TT vaccine	F: HIV PMTCT	G: Anti-Helmintic
**A: ITNs**		A (2)	A (3)	A (3)	A (3)	A (0)/F (0)	A (3)
**B: IPTp**			B (3)	D (2)	E (1)	B (0)/F (0)	B (2)
**C: IRS**				D (2)	E (3)	F (3)	G (2)
**D: Iron**					D (0)/E (0)	F (1)	D (1)
**E: TT vaccine**						F (1)	E (3)
**F: HIV PMTCT**							F (3)
**G: Anti-helmintic**							
**Total score per intervention**	**A: 14**	**B: 5**	**C: 0**	**D: 5**	**E: 7**	**F: 8**	**G: 2**

### Perceptions of IPTp

According to the IDIs, women perceived that the different drugs given at the ANC were to prevent diseases in general, but most (70%) could not distinguish them apart (neither by name nor by description) nor could mention the single health problem targeted by such drug. They used the term *comprimidos* (Portuguese term for “tablet”) to refer to those drugs and were aware that one of the tablets was given to prevent malaria, although very few could specify which tablet. Very few referred to it as *Fansidar* (Commercial name for SP in Mozambique). Therefore, after the initial open-ended question about the purpose of the different tablets given at ANC was answered, and in order to further explore the topic of IPTp, the interviewer referred to it as “the white tablets given to pregnant women at the ANC to prevent malaria and normally taken in front of the nurse”.

All participants attending ANC reported to have accepted to take the tablets given by the nurse. In terms of perceived side effects of such tablets, some reported dizziness, vomiting, malaise and nausea. The majority (59%) of the women who reported side effects attributed them to taking the tablets too early in the morning and on an empty stomach.

*“I felt normal after taking those tablets, but, because I left my house without taking matabicho (breakfast), I felt very weak”*. – 18 years old pregnant women, IDI1

For the majority of women (76%), the fact that the tablets were provided at the health facility made IPTp trustworthy. Other sources of tablets (such as informal vendors) were seen as dangerous for pregnant women.

### Reported use of malaria preventive strategies at home

When discussing home-based malaria preventive strategies, again the majority of the women (75%) reported sleeping under an ITN (Table 7), and those who did not use it claimed that it was due to difficulties in hanging the net. According to the women, ITNs were preferred because they provide protection against mosquitoes to more than one person at once, and that, unlike insecticides this protection is long-lasting.

**Table 7:** Reported use of malaria preventive measures among the interviewed women (n = 85)*.

Preventive tools at the household	Frequency mentioned (%)**
ITN	65 (77)
Insecticides (sprays or coils)	7 (8)
Burning of biomaterials *(Sule)*	6 (7)
Domestic hygiene (sweeping the backyard, cutting the grass, pruning the trees)	2 (2)
Insect repellent	2 (2)
IRS	1 (1)
None	2 (2)

* Responses from 1^st^ round of interviews.

** Participants were allowed to mention more than one strategy.

All participants were aware that because they were pregnant they had priority in receiving and using ITNs. Small children and their mothers were also perceived as priority groups.

*“If the decision depended on me, I would have priority in the use of mosquito net because I'm pregnant and I need to protect myself”*. 20 years old pregnant woman, IDI2

When probed about other methods they frequently used to protect against mosquitoes and malaria at home, including traditional ones, insecticide sprays, coils, and traditional smoke repellents were mentioned although much less frequently than ITNs. IRS was the least frequently mentioned malaria-preventive strategy adopted at home. Participants did not believe in the effectiveness of IRS, alleging that the chemical used “increased the burden of insects in the house, apart from not killing mosquitoes and others bugs”.

## Discussion

There is very limited information on what perceptions pregnant women exposed to malaria have about the infection, and what their acceptability to the recommended malaria preventive interventions is. With very few exceptions [25], specific studies on the acceptability of malaria preventive interventions tend to look at them in the context of clinical trials or examine such interventions in isolation from other interventions delivered through the same mechanism [26], for example ANC. This study adds to the body of evidence regarding prevention of malaria in pregnancy as an integral component of maternal and newborn health services.

The results of this study indicate that although the majority of pregnant women from this rural area in Mozambique recognize malaria infection as a health problem in pregnancy, in general they do not perceive it as dangerous for the pregnancy and the foetus. In this setting, where the high ANC attendance rate and the favourable delivery mechanisms for ITNs and IPTp offer potential to achieve the required coverage and use, there is a risk of the unwanted consequence of leaving pregnant women feeling not-at-risk. This finding calls for increased awareness raising about pregnant women's risk of malaria infection and its consequences in endemic areas where malaria continues to be an important health problem for pregnant women and children [27], [28], [29].

The observed limited awareness in this study regarding the adverse consequences of malaria in pregnancy, especially in relation to the foetus and the newborn, might contribute to low acceptability which in turn can negatively influence adherence, regarding malaria preventive interventions. For example, the low preference for IRS among pregnant women was evident in this study. A possible reason could be that the inconveniences and perceived ineffectiveness of the intervention could be superseding the perceived advantages of preventing malaria during pregnancy. This is in agreement with previously reported results from the same setting [30], [31]. This should be taken into account when defining malaria prevention recommendations for pregnant women in particular and the population as a whole.

ITNs were the highest ranked malaria preventive intervention by the pregnant women in this study. This finding offers an opportunity to improve the current coverage of ITNs in the households, which is still inadequately low in Mozambique (28%) [4], [32], by reinforcing ITN distribution through ANC services. This must be coupled with the provision, during the ANC health education sessions, of clear instructions and demonstrations on how to hang the ITN.

IPTp did not strongly feature as first choice by the women, and dominated the second and third choices compared to the other interventions. One-on-one Pairwise comparisons showed that among malaria preventive interventions, IPTp is preferable compared to IRS but neither prevails over ITNs, nor over most of the other ANC interventions. Provided that both IPTp and ITNs were rolled out to serve the same purpose of preventing malaria in pregnancy, the factors underlying preference of ITNs over IPTp should be critically examined. Again, convenience factors offered by ITNs, i.e., providing a physical barrier against the high burden mosquito bites [33], [34], may be playing a more important role in influencing acceptability and use, compared to the recognition of the threat of malaria in pregnancy.

Even though IPTp was not amongst the most preferred ANC intervention, there seemed to be adherence to it, as reported by study participants. In accordance, data from the DSS indicates that nearly 80% of women who delivered at the health facilities within the Manhiça Demographic Surveillance Area (DSA) had taken at least one dose of IPTp (unpublished data, Manhiça DSS 2010). In contrast to previous studies in Africa, which identified perceived adverse reactions to the drugs as a negative factor for adherence [8], there were no indications from this study that perceived adverse reactions could be negatively influencing adherence, since the participants who mentioned adverse reactions reported to still take the tablets. The administration of IPTp through the directly observed therapy may have favoured the reported adherence to this intervention. However, this is only applicable in settings like Manhiça District, where ANC attendance is high.

It has been shown that the nature of the provider does influence adherence to a given health intervention [8], [26], [35], [36]. In this setting, the hospital was viewed by the respondents as a “well-intentioned” entity, and their trust and obedience towards the health system, expressed by the term “the rule of hospital”, is consistent with findings from previous studies in the same setting [30], [37]. Interestingly, despite the presence of informal drug vendors and other sources of anti-malarial drugs in the study area, the study participants only trusted the formal health system for prescribing drugs to pregnant women. This positive behaviour should be reinforced and the existing awareness of the dangers posed by informally prescribed drugs should be transferred to other population groups.

A limitation of the study however, was the fact that part of the recruitment of participants as well as the first interviews with ANC attendants, took place at the health facility. This could have limited the level of comfort to openly discuss interventions provided by the health system. Involvement of pregnant women who had not attended ANC and conducting follow-up interviews at home contributed to the minimization of this social desirability bias [38]. However, community-based studies in similar settings are needed in order to provide supporting evidence regarding patients' judgement of the interventions.

A second limitation of the study rises from the assumption that preferences and ranking of interventions serve as proxies for acceptability. We acknowledge that these indicators cannot capture all dimensions of acceptability, but can be used as predictors of adherence to an intervention, taking into account its perceived relative importance over other interventions delivered through the same scheme. Future studies are recommended to explore further dimensions of acceptability of preventive interventions for malaria in pregnancy.

## Conclusion

In this malaria endemic area, low awareness among pregnant women of the risks and adverse consequences of malaria for pregnant women and neonates did not seem to affect acceptability or adherence to all of the recommended malaria preventive interventions in the same manner. Acceptability seemed to be intervention-specific and dependent on perceived convenience and other immediate benefits. The approach used for delivery, and the nature of the provider also played an influential role on adherence to the intervention. In the efforts to maximize overall coverage, pregnant women, through ANC services, can be the vehicles for ITN distribution in the community.

Malaria preventive interventions requiring community-based delivery mechanisms, such as IRS or drugs requiring self-administration, are expected to have a reduced adherence, unless the risks averted by the interventions are clearly understood by the target population. Therefore, improving the knowledge about the effects of malaria on maternal and neonatal health may help increase the acceptability and adherence to malaria preventive interventions, especially if delivered through channels other than the health facility.

## Supporting Information

Form S1 **In-depth Interview guide.**
(PDF)

Form S2 **Free-listing data extraction form.**
(PDF)

Form S3 **Pairwise comparison data extraction form.**
(PDF)
