# Supplementary material for: Perceptions of Malaria in Pregnancy and Acceptability of Preventive Interventions among Mozambican Pregnant Women: Implications for Effectiveness of Malaria Control in Pregnancy
Source: PLoS One. 2014 Feb 3;9(2):e86038. doi: 10.1371/journal.pone.0086038 (PMC3911904; doi:10.1371/journal.pone.0086038)
Supplement: Form S1 — In-depth Interview guide. (PDF) [file pone.0086038.s001.pdf]

cism centro de investigação em saúde de manhiça	**Centro de Investigação em Saúde de Manhiça**<br><br>**Estudo MIPPAD - Componente Social (MipSoc)**<br>**Guião de Entrevistas em Profundidade - participantes do ensaio clínico e mulheres da comunidade**<br>**V4**	ID no estudo: |__|__|__|__|-|__|__|__|__| ID na comunidade: |__|__|__|__|__| Entrevista Nº |__|__| Data: |__|__|/|__|__|/|__|__| Entrevistador: |__|__|__|

I. INSTRUÇÕES

1. Esta entrevista aborda questões relacionadas com a prevenção da malária durante a gravidez. Pretende explorar a percepção e o comportamento em relação à malária na gravidez, bem como a aceitabilidade e o uso de intervenções de prevenção de malária na gravidez oferecido nas unidades sanitárias.
2. Esta entrevista será feita a mulheres grávidas da comunidade e a mulheres que participam no ensaio clínico. Ajuste sempre as perguntas em função da pessoa entrevistada ser ou não participante do ensaio clínico.
3. Diga que você tenciona conversar sobre tópicos relacionados com a prevenção da malária na gravidez, mas que gostaria de falar desses tópicos informalmente, como se fosse uma conversa.
4. Este formulário está dividido em:

# Perguntas de pesquisa: Os temas que nós os investigadores queremos abordar.

Perguntas da entrevista: As questões que você como entrevistador fará directamente à participante para obter as respostas às questões de pesquisa.

5. *As instruções/sugestões ao entrevistar estão em itálico.*
6. O guião das entrevistas está dividido em 2 colunas.
   – **A coluna do lado esquerdo** contém os tópicos e abaixo as **perguntas da entrevista** com *algumas instruções e sugestões*. As perguntas da entrevista apenas servem para guiar a entrevista. A entrevista é aberta e em profundidade, o que significa que há necessidade de alguma improvisação até que a participante perceba a questão e consiga respondê-la. O rumo que entrevista tomar será diferente de entrevista para entrevista.
   – **A coluna do lado direito** é para você fazer um sumário das respostas. Faça o sumário geral das perguntas de pesquisa, e **NÃO** de cada uma das perguntas da entrevista. Estes sumários não devem ser limitados a um simples "sim" ou "não". Escreva algumas frases que respondem à pergunta de pesquisa. Os sumários não tem que ser demasiado detalhados, pois iremos encontrar os detalhes ao transcrevermos a entrevista. Tente preencher os sumários durante a entrevista. Se não tiver tempo suficiente durante a entrevista, termine o preenchimento imediatamente após o término da entrevista.
7. A entrevista terá que ser gravada, mas só se a participante concordar, caso não aceite, tome notas.

BOM TRABALHO!

Entrevistador(a) ☐☐☐ Data: ☐☐/☐☐/☐☐☐☐ ID no estudo (MIP-SOC): ☐☐-☐☐☐☐

II. IDENTIFICAÇÃO

Ficheiro Áudio: Arquivo nº: ☐☐

Ref. no computador:

☐☐☐☐-☐☐☐☐-☐☐

Perm_ID ☐☐☐☐-☐☐☐-☐☐☐-☐☐

Idade (anos) ☐☐

Tem filhos? ☐ Sim ☐ Não

Estado civil
☐ Solteira
☐ Relação sem compromisso
☐ Comprometida/casada/união marital
☐ Outro ___________________

Ocupação (descrever de acordo com a participante) ______________________________
____

Tipo de rendimento
☐ Sem rendimento
☐ Rendimento irregular
☐ Rendimento regular

Idade gestacional (meses) ☐

Local da entrevista ______________________________

Língua(s) falada(s) durante a entrevista ______________________________

Resultado da entrevista
☐ Completa
☐ Incompleta, razões _________________________
☐ Por completar a ☐☐/☐☐/☐☐☐☐

Conhecimentos de doenças em geral

**1. Que conhecimentos tem sobre as doenças no geral?**<br>• Quais são as doenças mais frequentes em adultos nesta zona? Quais são os sintomas dessas doenças e como se transmitem?<br>• Onde e com quem ouviu essa explicação?<br>• Existem algumas doenças que são mais comuns em certas épocas do ano? Quais são?<br>• Existem doenças que afectam principalmente mulheres grávidas? O que as pessoas normalmente pensam sobre as doenças em mulheres grávidas?	

Percepção e Conhecimentos sobre a malária

**2. Que percepção e conhecimentos tem sobre a malária?**<br>• Pode dizer o que sabe sobre a malária?<br>→ *Explorar os seguintes aspectos:*<br>– *Transmissão*<br>– *Prevenção*<br>– *Cura/Tratamento*<br>– *Sintomas*<br>– *Causas*<br>– *Consequências/Efeitos*<br>• E acredita que a malária se transmite assim (Cite as formas de transmissão que a participante mencionou mais acima)?<br>• Onde e com quem ouviu essa explicação?<br>• Se não houver mosquitos não haverá malária?	**SUMÁRIO**

• Como uma pessoa percebe que tem malária?<br>• O que acontece depois da picada do mosquito? Como o mosquito provoca a malária?	

Risco de contrair malária na gravidez

**3. Que percepção tem sobre o risco de contrair malária durante a gravidez?**	**SUMÁRIO**
• Acha que há grupos mais vulneráveis do que outros a apanhar a malária? Porquê?<br>• Quais são as doenças mais frequentes em mulheres grávidas?<br>• Que doenças tornam as mulheres grávidas mais susceptíveis a apanhar malária? (*caso mencione alguma procure saber porquê*).<br>• Sente-se em risco de ser infectada pela malária? Porquê?<br>• A malária nas mulheres grávidas, actua da mesma maneira como em outras pessoas?<br>• Como se sente uma mulher grávida quando tem malária? O que pode acontecer com ela? E com o bebé?<br>• Actualmente está usar algum método de prevenção contra a transmissão da malária? Porque? Porque não?<br>*→ Caso sim, questionar a frequência de uso; (sempre ou às vezes) por quanto tempo acha que irá usar esse (s) método (s). Explorar se sente que está a proteger a si própria e/ou aos outros; Explorar também a proveniência da recomendação para o uso.*	

Percepção do Serviço do SMI (Saúde Materno Infantil)

<table>
<tr>
<td>

4. Percepção e conhecimentos sobre o SMI

- Já começou com a consulta pré-natal? Acha que foi cedo/tarde? O que lhe motivou a ir a consulta pré-natal?
- Se não quando pensa começar?
- Com quantos meses de gravidez acha que se deve ir à primeira consulta pré-natal?
- Em que medida considera útil a consulta pré-natal? Sabe para quê serve a ficha? Concorda que esta consulta seja obrigatória?
- Como se chama o local onde fazem a consulta pré-natal?
- Costuma conversar sobre o que acontece na consulta pré-natal com seu parceiro e com outras mulheres? Com os seus familiares?
- O que é que se faz nesta consulta? Concorda com os procedimentos?
- Durante a consulta deram algum medicamento? O que foi que lhe disseram sobre estes comprimidos no hospital? (Se não, passa para a pergunta seguinte)

→ *Se sim, explorar que medicamentos são, para que disseram que servem, como se tomam e durante quanto tempo se toma, se teve alguma dificuldade na toma desses comprimidos. Explorar ainda se teve algum problema depois da toma e como se sentiu quando tomou os comprimidos.*

- Acha que é aceitável que as mulheres grávidas tomem estes comprimidos?

</td>
<td>

SUMÁRIO

</td>
</tr>
</table>

Estratégias de prevenção

<table>
<tr><td>

5. Estratégias de prevenção da malária

Quais são os diferentes métodos de prevenção da malária que conhece? Dos métodos que mencionou quais são os mais aceitáveis e eficazes? Qual dos métodos esta a usar? E como escolheu o método de prevenção a usar? (Se não usa nenhum explorar as razões).

→ *Se mencionar que usa rede mosquiteiras explorar:*

– *Porque esta a usar a rede? Como está a usar?*
– *Há quanto tempo e com que frequência usa?*
– *Quem dorme na rede? Quem decidiu isso?*
– *Como se sente dormindo na rede? Existe alguma coisa que não gosta quando dorme por baixo da rede?*
– *Acha que a rede lhe protege da malária? Porque sim? Porque não?*
– *Onde arranjou a rede? (se comprou acha que foi caro)?*

- Existe alguma dificuldade no acesso as redes? Qual?
- Teve alguma dificuldade a montar a rede?
- O que fazia para se proteger antes de ter rede? (*Explorar como é que os outros em casa fazem para se protegerem*) Se a escolha dependesse de si, quem teria priorid*ade de usar a rede mosquiteira caso houvesse apenas uma rede* na sua casa? Porquê?

→ *Se mencionar que não usa rede mosquiteira explorar porque não tem rede mosquiteira?*

</td><td>

<u>SUMÁRIO</u>

</td></tr>
</table>

Percepção da prevenção da malária *na* gravidez

6. O que percebe da prevenção da malária<br>• Conhece algum medicamento que se toma para a redução do risco de contrair malária durante a gravidez? Qual?<br>• Como actuam? Quantas vezes tomam-se os comprimidos?<br>• Que vantagens e desvantagens podem trazer?<br>• Notou algum efeito depois de tomar os comprimidos?<br>• Que opinião tem a respeito destes comprimidos? Acha importante tomar estes medicamentos?<br>• Já tomou algum desses medicamentos?<br>• É bom tomar medicamentos durante a gravidez? Porque sim ou porque não?<br>• Em que altura a mulher deve evitar tomar medicamentos (ex: logo de manhã, logo depois de vomitar, com a barriga cheia, com a barriga vazia, quando acaba de tomar outros medicamentos do hospital ou tradicionais)?	

Aceitabilidade e uso de diferentes intervenções de prevenção da malária na gravidez

**7. Aceitabilidade das intervenções de prevenção de malária na gravidez**<br>• O que pensa sobre darem comprimidos para a prevenção da malária a mulheres grávidas? É aceitável?<br>• Há algum medicamento que as mulheres grávidas devem evitar tomar? (Explorar qual medicamento e porque não devem tomar).<br>• Que opinião tem sobre o tratamento?<br>• Se lhe provocassem alguns sintomas, o que teria pensado em fazer?	**SUMÁRIO**

• Depois de tomar os comprimidos apareceu algum sintoma?<br><br>→ *Se sim, explorar:*<br>– *Quais? Qual era a intensidade?*<br>– *Quando começou a notar esses sintomas?*<br>– *Porque razão acha que os sintomas apareceram?*<br>– *Tomou alguma medida para resolver? Que medida? (Se não passe para a pergunta seguinte)*<br>– *A situação se manteve durante quanto tempo?*<br>– *Como se sentiu em relação a esses sintomas? E pensou em desistir de tomar os comprimidos?*<br>– *Recebeu algum conselho sobre como agir? Que tipo de conselhos recebeu e de que? (Se não passe para a pergunta seguinte)*<br>– *Esperava ter tido esse tipo de sintomas? Porque?*<br>• Acha que é importante seguir o tratamento ou não? Porque/porque não?<br>→ *Se a participante não faz parte do ensaio clínico PASSE PARA A PERGUNTA 9.*	

Conhecimentos sobre o estudo (Só para participantes do ensaio clínico)

**8. O que as participantes sabem sobre o estudo MIPPAD?**<br>• Sabe de que assunto trata este estudo?<br>• Qual é a finalidade do estudo? (*explorar a informação que ela teve através do Consentimento Informado, sobre os requisitos para participar, de que se trata, onde decorre, requisitos para participar e os procedimentos do estudo*).	

• Acha que o estudo é importante? Porque?<br>• Toda informação dada durante a explicação do CI foi suficiente para tomar a decisão de participar no estudo? Não se sentiu pressionada para participar do estudo?<br>• Quando entrou para tomar os comprimidos o que foi que lhe disseram?<br>• O que lhe motivou a participar do estudo?<br>• Acha que há algum benefício em participar do estudo?<br>• Depois de ter tomado os comprimidos como se sentiu? Teve algum sintoma?<br>• O que pensou depois de tomar os comprimidos? Acha que depois de tomar estes comprimidos nunca mais vai apanhar malária?<br>• Como reagiram os comprimidos?<br>• Para participar no estudo foi obrigada?<br>• Deram-lhe tempo para pensar? Se lhe dessem tempo para pensar a quem perguntaria na sua casa?<br>• Quem sabe que esta a participar neste estudo?<br>• O facto de tomar comprimidos para a prevenção da malária fez com que prestasse mais atenção no uso de estratégias de prevenção da malária?	

Poder de decisão

**9. Que poder de decisão tem as mulheres em relação ao uso de diferentes estratégias de prevenção da malária na gravidez?**	**SUMÁRIO**
• Decidiu sozinha participar no estudo? Decidiu ali no hospital? O seu parceiro sabe que participa no estudo?<br>• Se lhe dessem tempo para pensar a quem consultaria em casa?<br>• Quem mais sabe que está a participar no estudo? Essa pessoa influenciou na sua decisão?<br>• Teve tempo para pensar ou teve que decidir na hora?<br>• Qual é a sua opinião sobre este estudo?<br>• As mulheres grávidas têm aceite participar neste estudo? Porque acha que sim/porque acha que não?	

10. Notas/Observações

____
____
____
____
____
____
____
____
____
____
____
____
