# Supplementary material for: Perceptions of Malaria in Pregnancy and Acceptability of Preventive Interventions among Mozambican Pregnant Women: Implications for Effectiveness of Malaria Control in Pregnancy
Source: PLoS One. 2014 Feb 3;9(2):e86038. doi: 10.1371/journal.pone.0086038 (PMC3911904; doi:10.1371/journal.pone.0086038)
Supplement: Form S2 — Free-listing data extraction form. (PDF) [file pone.0086038.s002.pdf]

<table>
<tr>
<td></td>
<td>Centro de Investigação em Saúde de Manhiça<br><br>Estudo MIPPAD – Componente Social do estudo (MipSoc)<br>Listagem livre de doenças durante a gravidez<br>V2</td>
<td>ID no estudo: |__|__|__|__|__|__|<br>Entrevista Nº |__|__|<br>Data: |__|__|/|__|__|/|__|__|<br>Entrevistador): |__|__|__|</td>
</tr>
</table>

## Procedimentos

1. Perguntar sobre doenças no geral em mulheres grávidas (que doenças as mulheres grávidas costumam a ter?), caso alguma mencione algum sintoma, perguntar a que doença esse sintoma está ligado. Comece por preencher a primeira coluna.
2. Depois de preencher a primeira coluna, comece pela primeira doença e pergunte que sintomas e sinais costumam ter as mulheres grávidas com está doença. Anote as respostas na coluna 2.
3. Pergunte qual é o principal sintoma e assinale com um círculo ou sublinhe esse sintoma.
4. Pergunte qual é a causa da doença. Preencha a resposta na coluna 3.
5. Pergunte qual é o tratamento (falar de qualquer tipo de tratamento e procurar saber como exactamente fazem com esse tratamento). Anote a resposta na última coluna.
6. Repita os passos 2 a 5 para cada doença mencionada (ou se a participante ficar cansada, tente trabalhar apenas com 5 doenças).

| Nome da doença em mulheres grávidas | Sintomas e sinais (sublinhe ou circule o principal) | Causas | Tratamentos |
|---|---|---|---|
| | | | |
| | | | |
| | | | |
| | | | |
| | | | |
| | | | |
| | | | |
| | | | |
| | | | |

| Nome da doença em mulheres grávidas | Sintomas e sinais (sublinhe ou circule o principal) | Causas | Tratamentos |
|---|---|---|---|
| | | | |
| | | | |
| | | | |

**Notas/Observações**

______________________________________________________________________________
______________________________________________________________________________
______________________________________________________________________________
______________________________________________________________________________
______________________________________________________________________________
______________________________________________________________________________
______________________________________________________________________________
______________________________________________________________________________
______________________________________________________________________________
______________________________________________________________________________
______________________________________________________________________________
______________________________________________________________________________
______________________________________________________________________________
______________________________________________________________________________
