# Supplementary material for: Perceptions of Malaria in Pregnancy and Acceptability of Preventive Interventions among Mozambican Pregnant Women: Implications for Effectiveness of Malaria Control in Pregnancy
Source: PLoS One. 2014 Feb 3;9(2):e86038. doi: 10.1371/journal.pone.0086038 (PMC3911904; doi:10.1371/journal.pone.0086038)
Supplement: Form S3 — Pairwise comparison data extraction form. (PDF) [file pone.0086038.s003.pdf]

cism centro de investigação em saúde de manhiça	**Centro de Investigação em Saúde de Manhiça**<br><br>**Estudo MIPPAD - Componente Social do estudo (MipSoc)**<br>**Comparação aos pares de estratégias de prevenção de malária na gravidez e intervenções do SMI**<br>**V4**	ID no estudo: |__|__|__|__|-|__|__|__|__|-|__|__|__| Data: |__|__|/|__|__|/|__|__| Entrevistador: |__|__|__|

1. Intervenções ao nível da Unidade Sanitária

Mostre os cartões com as figuras, explicando que cada cartão representa uma forma de prevenção de doenças e pergunte a participante se para além daquelas medidas, se ela leva a cabo outras medidas de prevenção para qualquer outra doença ou problema de saúde (incluindo estratégias caseiras) e anote estas estratégias em cartões de reserva. Peça que a participante escolha a ordem de preferência das estratégias de prevenção que ela prefere usar durante a gravidez. Usando uma escala de 1 a 7, preencha a segunda coluna com as opções por ela escolhidas. Repita este processo até acabarem todas as figuras. Pergunte pela razão da preferência de cada opção escolhida e anote na última coluna.

**Estratégias de prevenção**	**Ordem de preferência**	**Razão da preferência**
Rede mosquiteira (Malária)		
Fansidar (malária)		
Pulverização intra domiciliária (malária)		
Sal ferroso (Anemia)		
Vacina (Tétano)		

Estratégias de prevenção	Ordem de preferência	Razão da preferência
PTV (HIV)		
Mebendezol (Parasitoses)		
Outra opção ____________________________ ____		

2. Comparação aos pares de estratégias caseiras de prevenção da malária na gravidez e intervenções ao nível da Unidade Sanitária

Disponha as figuras aos pares de acordo com a tabela abaixo. Para cada par pergunte qual dos dois procedimentos ela prefere usar. Anote a resposta dada na terceira coluna (coloque o número 1 ou 2 de acordo com a resposta). Depois, pergunte pela razão da preferência de cada opção escolhida e anote na última coluna. Repita este processo até acabarem todos os pares de opções.

Pares de estratégia de prevenção		Preferência	Razão da preferência
1.Rede mosquiteira	2.Mebendazol		
1.Fansidar	2.PIDOM		
1.Sal ferroso	2.Vacina		
1.Mebendazol	2.PTV		

Pares de estratégia de prevenção		Preferência	Razão da preferência
1.PIDOM	2.Sal ferroso		
1.Vacina	2.Rede mosquiteira		
1.PTV	2.Fansidar		
1.Sal ferroso	2.Mebendazol		
1.Vacina	2.PTV		
1.Rede mosquiteira	2.PIDOM		
1.Fansidar	2.Vacina		
1.Sal ferroso	2.Rede mosquiteira		
1.PIDOM	2.PTV		

Pares de estratégia de prevenção		Preferência	Razão da preferência
1.Mebendazol	2.Fansidar		
1.Vacina	2.PIDOM		
1.Rede mosquiteira	2.Fansidar		
1.Sal ferroso	2.PTV		
1.PIDOM	2.Mebendazol		
1.Fansidar	2.Sal ferroso		
1.Rede mosquiteira	2.PTV		
1.Mebendazol	2.Vacina		

3. Notas/Observações

____

____

____

____

____
